# National surveillance using mobile systems for health monitoring: complexity, functionality and feasibility

**DOI:** 10.1186/s12879-019-4338-z

**Published:** 2019-09-16

**Authors:** Yages Singh, Debra Jackson, Sanjana Bhardwaj, Natasha Titus, Ameena Goga

**Affiliations:** 1 Health Systems Research Unit, South African Medical Research Council, Cape Town, South Africa; 2 School of Public Health, University of the Western Cape, Cape Town, South Africa; 3 Health Section, UNICEF, New York, USA; 4 Health Section, UNICEF South Africa, Pretoria, 0001 South Africa; 5 Department of Paediatrics, University of Pretoria, Pretoria, South Africa

**Keywords:** Technology, Mobile phones, Data collection

## Abstract

**Background:**

Although the use of technology viz. mobile phones, personalised digital assistants, smartphones, notebook and tablets to monitor health and health care (mHealth) is mushrooming, only small, localised studies have described their use as a data collection tool. This paper describes the complexity, functionality and feasibility of mHealth for large scale surveillance at national and sub-national levels in South Africa, a high HIV-prevalence setting.

**Methods:**

In 2010, 2011–12 and 2012–13 three nationally representative surveys were conducted amongst infants attending 580 facilities across all 51 districts, within all nine provinces of South Africa, to monitor the effectiveness of the programme to prevent mother-to-child transmission of HIV (PMTCT). In all three surveys a technical protocol and iterative system for mobile data collection was developed. In 2012–13 the system included automated folders to store information about upcoming interviews. Paper questionnaires were used as a back-up, in case of mHealth failure. These included written instructions per question on limits, skips and compulsory questions. Data collectors were trained on both systems.

**Results:**

In the 2010, 2011–12 and 2012–2013 surveys respectively, data from 10,554, 10,071, and 10,536 interviews, and approximately 186 variables per survey were successfully uploaded to 151 mobile phones collecting data from 580 health facilities in 51 districts, across all nine provinces of South Africa. A technician, costing approximately U$D20 000 p.a. was appointed to support field-based staff. Two percent of data were gathered using paper- questionnaires. The time needed for mHealth interviews was approximately 1,5 times less than the time needed for paper questionnaires 30–45 min versus approximately 120 min (including 60–70 min for the interview with an additional 45 min for data capture). In 2012–13, 1172 data errors were identified via the web-based console. There was a four-week delay in resolving data errors from paper-based surveys compared with a 3-day turnaround time following direct capture on mobile phones.

**Conclusion:**

Our experiences demonstrate the feasibility of using mHealth during large-scale national surveys, in the presence of a supportive data management team. mHealth systems reduced data collection time by almost 1.5 times, thus reduced data collector costs and time needed for data management.

## Introduction

The use of technology in research has grown rapidly in recent years in both developed and developing countries. Key considerations when planning data collection include the data collection method, the efficiency of the data collection process and the accuracy of the data collected [1]. The collection and management of research data is challenging; consequently choosing a simple, effective, cost efficient tool which produces accurate data is essential for success.

Electronic Data Collection systems (EDC) using modern technology has fast penetrated the research world. This increased use of technology has also included m-Health, a component of electronic health which refers to the use of mobile communication technologies to support healthcare practices (e.g. health data collection, delivery of healthcare information or patient observation and provision of care) [2]. Various technological devices such as basic mobile telephones, personalised digital assistants (PDAs), smartphones, notebook and tablets, have been used for data collection. The emergence of mobile devices including smart phones, with a host of integrated innovative features such as creating and receiving information, electronic signing and data collection software has opened the door to a new generation of measurement tools for data collection [3]. Recent changes in their functionality and portability have increased the potential utility of mobile technologies for research data collection [4]. Mobile data collection (MDC) is the gathering of structured information using mobile devices thereby revolutionising the way face to face surveys are approached and data are managed [5]. Traditionally, data collection was conducted using paper-based forms and data were subsequently double entered into a database to create electronic records [6]. Although this practice is well established, it is time and resource consuming, as well as error prone [6]. Common errors may be due to an inability to read the data collectors’ handwriting [7]. The financial and opportunity costs associated with data entry, double entry and storage are generally high. There is increasing evidence that digital data collection is feasible, faster, often more reliable, user-friendly and more economical than traditional data collection methods [8–11]. The major advantages of EDC is the ability to enter and review data, implement online data validation checks for more effective data quality assurance, monitor data collection progress, and analyse data, all in real time [6]. Additionally, EDC systems provide opportunity for daily, remote quality control and supervision of field-based data collectors which makes it an attractive management tool and preferable to a pen and paper-based approach [7]. The access to real time data enables better decision making for researchers, yielding faster results that will influence program action and/or policy making decisions and improved allocation of limited financial resources [7, 8].

To date studies that have reported MDC as a feasible option have been relatively small-scale surveys [2]. Large scale nationally representative surveys have traditionally used paper-based tools, necessitating secondary steps of data entry and management after data collection [12]. Consequently, errors occurring during data collection or data entry may not be rapidly identified [12]. There are several advantages of using MDC for large-scale surveys. [4–11] The use of mobile data collection systems for national surveillance studies has not previously been reported. This paper describes the complexity, functionality and feasibility of using mHealth for national surveillance.

## Methods

The South African PMTCT evaluations were nationally representative cross-sectional facility-based surveillance studies conducted between June–December 2010, August 2011–March 2012 and October 2012–May 2013. A cohort study was conducted between November 2012 and September 2014 among infants identified as being HIV exposed at 6 weeks during the 2012–2013 cross sectional survey. Data collection included interviews from consenting caregivers, record reviews and infant dried blood spots (iDBS) specimens to identify HIV-exposed infants (HEI) and HIV-infected infants. The 2012–2013 survey was a cross-sectional survey with a complex longitudinal observation component that followed up infants until 18 months post-delivery (until September 2014). Through a tender process, a South African-based digital company, Mobenzi, was appointed to develop a system for the collection and management of data collected using mobile phones. Catering for the needs of the study and the SAPMTCT team, the web based service provider, Mobenzi, developed a technical protocol that guided the development of the various mobile applications needed for the successful implementation of the study. Three data sources (interview/record review data, laboratory data and consent form data) were merged for final analysis.

### Technical background

#### Developing the mHealth data collection instrument and platforms

**Fig. 1:**
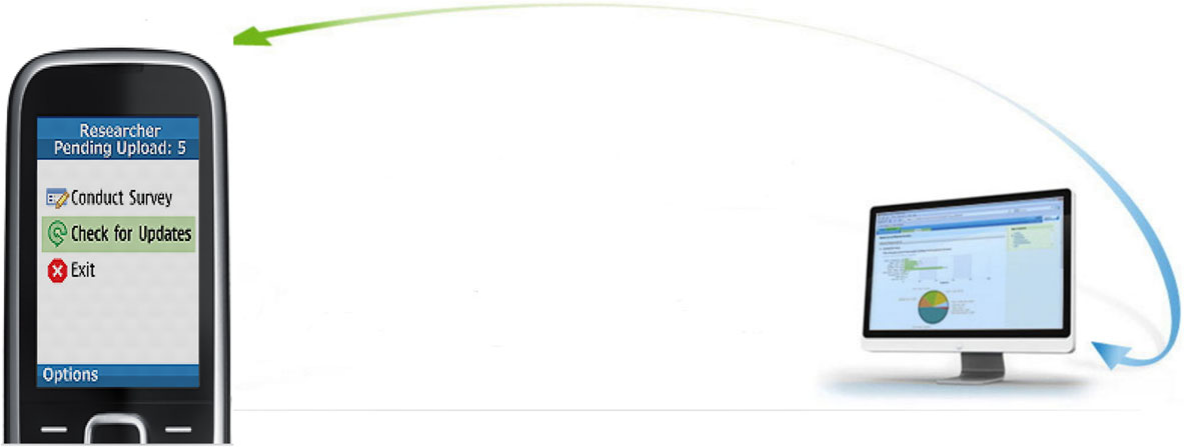
Mobile phone interface

Mobenzi was tasked with developing a user-friendly system to aid data collection, monitor and facilitate supervision of a large cadre of field based data collectors and ensure safe and secure data transmission and storage through all these processes. The system was designed to integrate the activities of data collectors into a web based interface that was initiated the moment a participant was recruited. Mobenzi was responsible for converting the MS Word based survey to an application suitable for implementation via mobile phones. The developed software applications were installed in low cost mobile phones (Nokia 2330) and data collected was uploaded onto a secure, password protected web based platform in real-time (Fig. 1).

#### Developing the mHealth technical protocol

Two customised platforms, Mobenzi Researcher that supported the data collection component and Mobenzi Outreach that supported the operational back-end functioning of the system were developed.

**Fig. 2:**
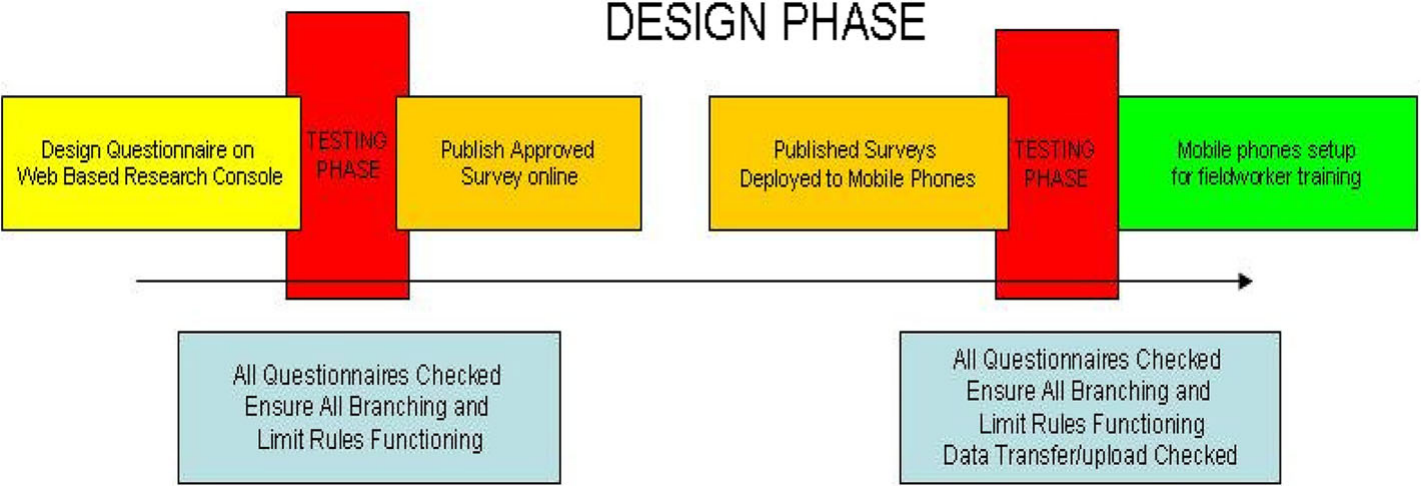
Technical protocol design phase

The developed software comprised five sets of electronic survey questionnaires: 1) 6-week recruitment questionnaires, 2) post-natal follow up questionnaires, 3) participant contact information form, 4) suspension/terminations and 5) missed visits forms. This multitude of survey questionnaires needed to be captured at various stages of the study. Customised visit timepoint folders with icons were developed and electronic questionnaires were housed in titled folders relevant to the visit at the time of participant interaction. This feature allowed for a user-friendly interface for data collectors, who were mainly retired nurses. Regardless of the complexity of the questionnaire or survey, data collectors could respond to each instruction one-by-one. Mobenzi Researcher handled extensive branching, skip logic and validations. Once a data collector accesses the folder relevant to that visit type, the research questionnaire is deployed in minutes ready for data collection Fig. 2.

#### Implementing the mHealth technical protocol

Each data collector was given their own study mobile phone and each phone was assigned to a fixed province (in which the data collector worked) and to a fixed number of health facilities sampled in that province for data collection. This ensured that data collectors would not randomly choose facilities that were not sampled for this study. The store and forward capability of the mobile software application enabled data collectors to continue data collection even without network connectivity. Collected data could be stored locally until a network reception was available and data was uploaded automatically onto the web based console. Data captured on the phones were protected with a write-only security model, encoded and stored in the Mobenzi management system. A multi-layered security protocol supported by the system ensured a high degree of participant confidentiality throughout the study. This feature ensured that only users of the Mobenzi researcher application, who had password controlled access, could access the data.

Data collectors could modify and review data while the interview was in progress. Any changes to questions and reminders to data collectors were sent via bulk SMS notification system to all users indicating that a change has taken place and users must check for updates on the mobile phone application. Mobenzi automatically loaded a set amount of airtime at month end for the duration of the study. Airtime was used for the uploading of data collected, to contact participants to set up appointments and to contact study team members when necessary.

#### The mHealth system used for the cross-sectional six-week component

**Fig. 3:**
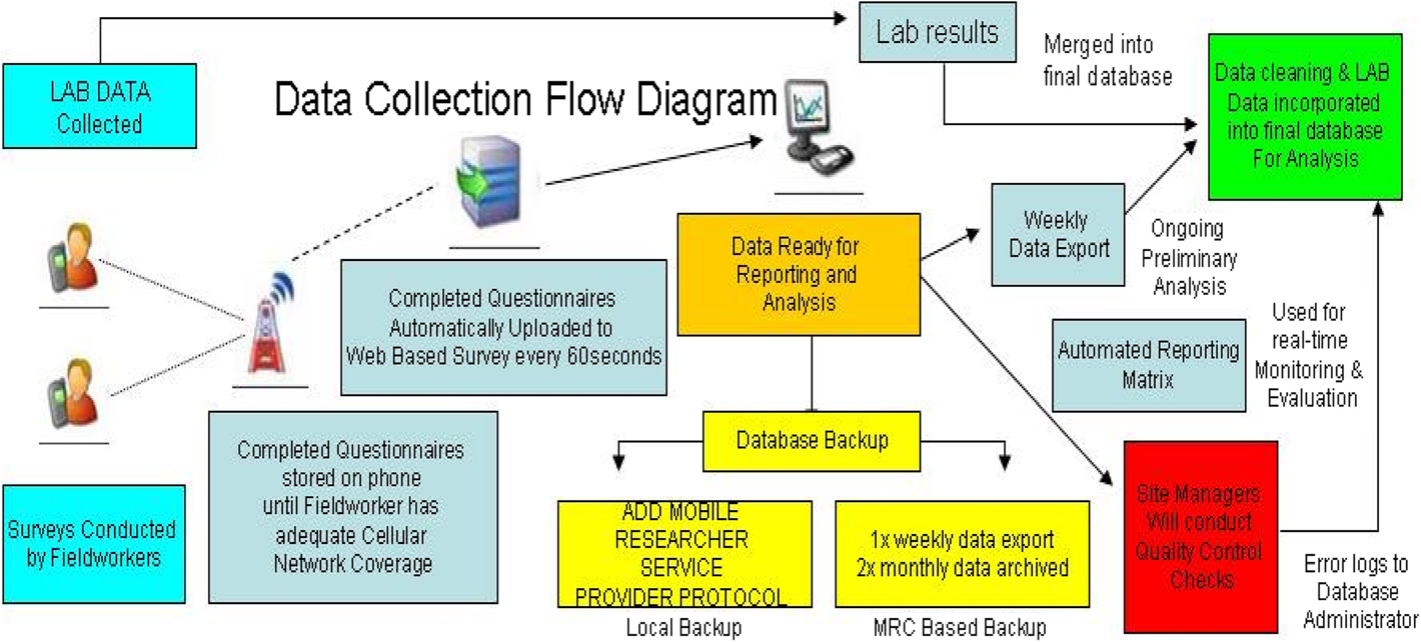
Data Collection flow diagram

The mobile system contained a pre-interview screening questionnaire which guided data collectors through the eligibility and consent processes, reminding them of eligibility criteria for the study (infants 4–8 weeks of ages, receiving their six-week immunisation and not needing emergency care) and of the consent procedures. At the recruitment stage (6-week visit), consented mothers/caregivers of infants were assigned a unique Participant Identifier (PID). The mother/caregiver was interviewed and an infant dried blood spot (DBS) was drawn. Consent for interview, infant blood draw, follow-up and use of infant’s DBS was captured on paper-copies and electronically. For participants who consented to being contacted for follow-up a participant contact information form was captured electronically, unlinked to the questionnaire, and uploaded for storage in a separate password protected cloud. Infant blood specimens were assigned a unique laboratory tracking number and unnamed results, identified only by the unique participant identifier, the laboratory tracking number, the date of visit and the visit time point were e-mailed to Mobenzi for merging into the raw dataset. During merging the PID, Lab tracking numbers, date of interview and visit type was matched with the same information in the raw dataset Fig. 3.

#### The mHealth system used for the follow-up component

**Fig. 4:**
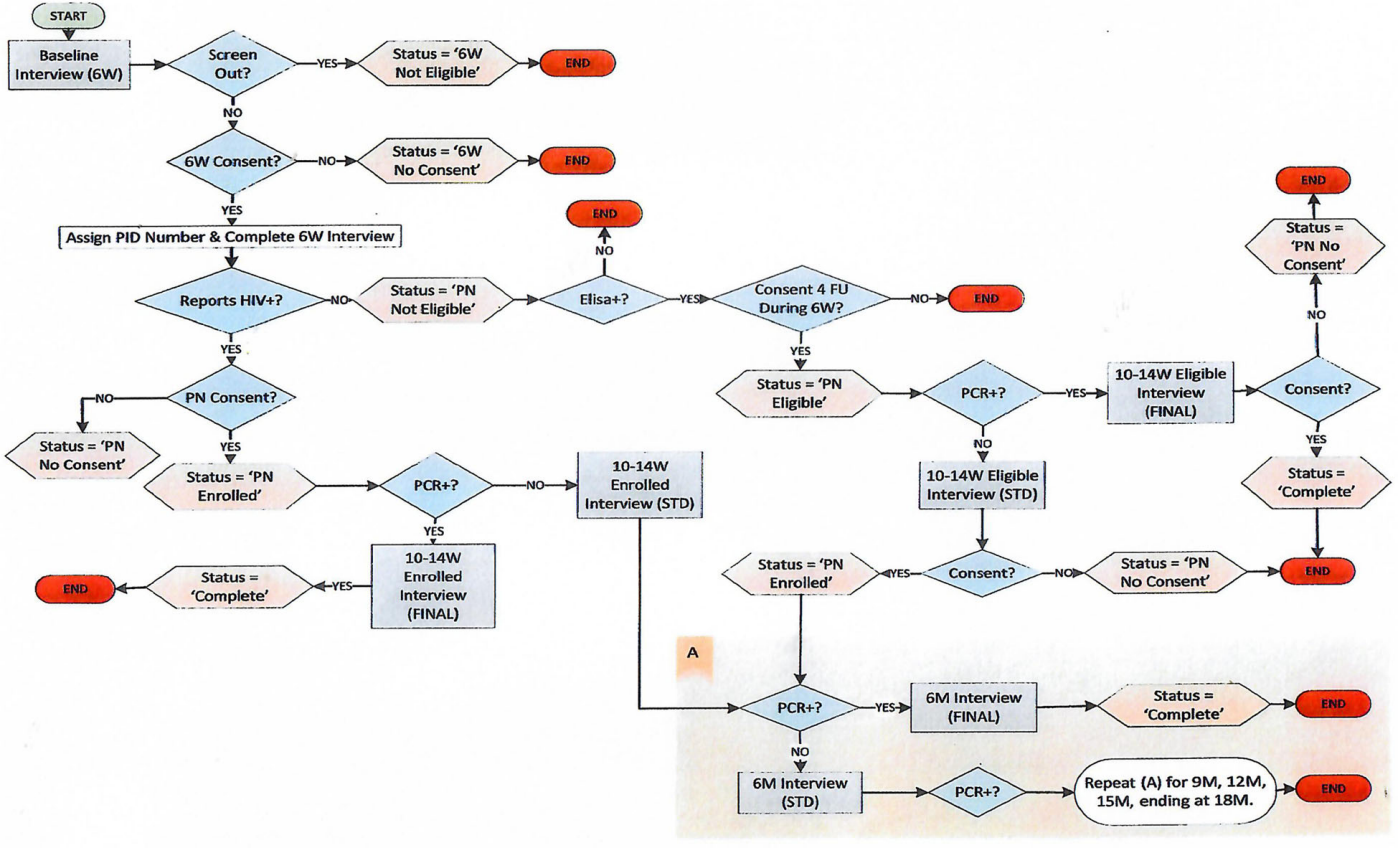
Operational workflow from Mobenzi platform

**Fig. 5:**
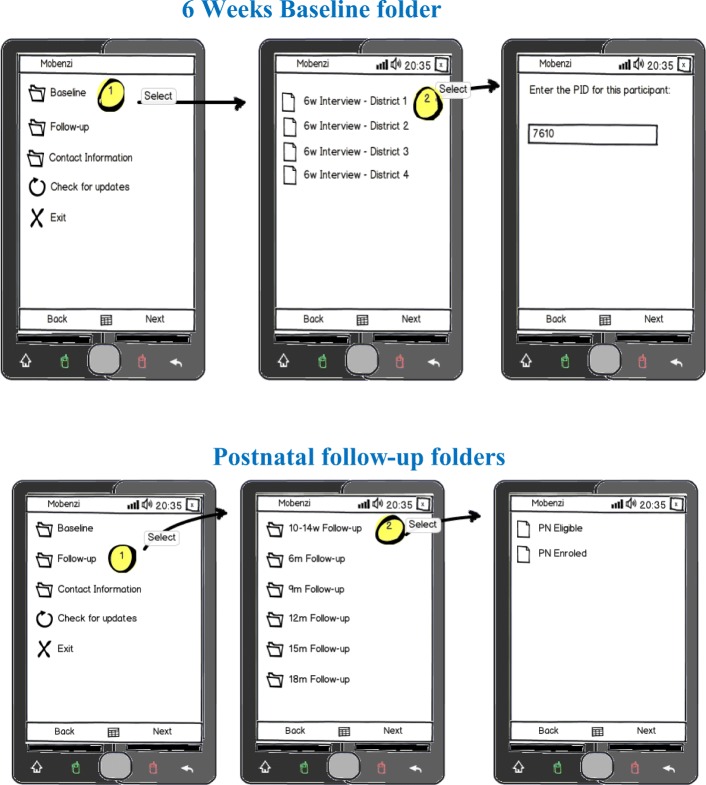
View of mobile phone interface for 6wks baseline and follow up visits

During the 2012–14 follow-up component the bi-directional web-based console enabled data collectors to request a confidential participant contact information form to facilitate follow-up of their allocated participants. Eligible HIV-exposed uninfected infants were invited for follow up at three, six, nine, twelve, fifteen and eighteen months. The eligible mother/caregiver infant pairs PIDs were pre-assigned and automatically appeared in the appropriate folder of the assigned data collectors study phone. If an infant was PCR negative, the system stored that PID and automatically triggered the next scheduled follow up visit (see operational workflow Fig. 4). Upon completion of that scheduled visit, that PID automatically moved to the folder of the next follow up visit Fig. 5. If an infant PCR results was positive, the system automatically stored these PIDs in a separate exit interview folder triggering an exit interview reminder to the data collector at the next scheduled visit. Three automated SMS reminder service was delivered to caregivers who provided contact information at the following timepoints:

- At 4 weeks after completing the baseline assessment at recruitment: *"Please return to the clinic when your baby is 10 weeks old for your baby’s check-up and 10-week immunisation*.
- Four weeks after each non-final follow up interview*: It has been 4 weeks since your babies last check. Please remember to return to the clinic for your baby’s check-up*.
- On enrolment into the postnatal study: *Thank you for enrolling in the MRC follow up study with your baby. Your nurse data collector will contact you within the next 2 to 3 weeks to make an appointment.*

The electronic system tagged for suspension of four groups of participants, namely, participants who could no longer be contacted or had missed more than three consecutive visits, or who refused interviews after initially consenting to follow-up and finally babies who had died. These tagged suspensions appeared in a folder accessed by the Data manager, for approval. Once a participant’s suspension was approved on the system, that participant identifier number was automatically removed from the list of follow up participants and no longer appeared in the relevant data collector’s mobile device folder. If a participant moved or relocated to another province and still wished to remain in the study, the Mobenzi system allowed for the participant to be transferred to another facility and the relevant PID was linked to the data collector assigned to that province and facility.

### Training data collectors on mHealth

The entire study team (data collectors, their supervisors, research support staff, co-and principal investigators) participated in a 5-day training including a one-day workshop conducted by the service provider on the use of the mobile phone data collection including, how to access the software application, checking for updates, accessing visit folders and how to administer the survey questionnaire. The training was conducted according to the standardised manual of operating procedures (SOPs), and addressed how to collect data using the mobile data collection tool and paper-based questionnaires. Training was also followed by several other 1 hour on-research-site refresher courses throughout the duration of the project. Weekly face-to-face supervision group meetings were held, with individual supervision when necessary. Data collectors could access daily telephonic support, provided by the SAMRC data management team. Data collectors were also trained on how to use a paper-based questionnaire in times of mobile system failure.

### Supervision using mHealth systems and web-based systems

Supervisors used the web-based interface to monitor and mentor data collectors remotely in between face to face supervisory visits. Additionally, the Mobenzi system generated automated daily and weekly summary reports that were emailed to supervisors and other designated staff. The automated report supported the management of the daily workflow/output, the name of data collector, the facility were data was collected, the date and time of interview, the PID, if DBS was drawn and if participant consented. Table 1.

**Table 1:** Screenshot of daily summary report

DC Name	Facility	Time Interview Start	Time Interview End	Date of Visit	Screen ID	PID	6w Eligible	6w Consent	6w DBS	PS Eligible	PS Consent
xxxx	kz Chatsworth Township Centre Clinic	09:00:08	10:08:46	2012-10-29	06541	341,856	0	0	0	0	0
xxxx	kz Mpumalanga Clinic	09:46:54	10:24:32	2012-10-29	05893	341,659	0	0	0	0	0
xxxx	kz Mpumalanga Clinic	09:55:59	12:15:40	2012-10-29	05894	341,343	1	1	1	0	0
xxxx	kz Chatsworth Township Centre Clinic	10:04:06	12:30:54	2012-10-29	06542	340,573	1	1	1	1	1
xxxx	kz Mpumalanga Clinic	09:44:39	11:21:21	2012-10-29	05891	341,341	1	1	1	1	1
xxxx	kz Inanda C CHC	12:32:12	01:24:20	2012-10-29	06931	340,397	1	1	1	1	1
xxxx	kz Chatsworth Township Centre Clinic	12:40:28	01:57:50	2012-10-29	06543	340,574	1	1	1	0	0

^xxxxxx^Data collector’s names blocked out to maintain confidentiality

**Fig. 6:**
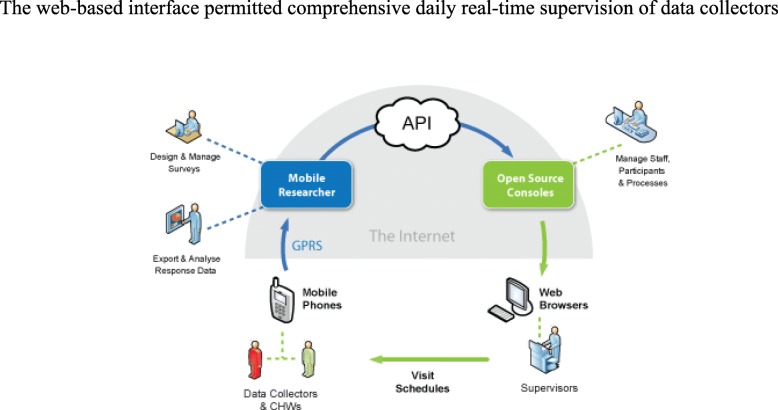
API: Application Programming Interface

Data collectors submitted daily work logs to supervisors who correlated this with the electronic reports and the scheduled workplan. (Table 1 and Fig. 6).

The web-based interface permitted comprehensive daily real-time supervision of data collectors.

### Data management

Completed questionnaires from the cross-sectional and longitudinal studies were uploaded from the mobile device to a web-based console, automatically and immediately. This ensured the confidentiality of the data collected i.e. if the mobile device is lost or stolen, only the blank questionnaires could be viewed. After questionnaires were uploaded onto the research console, they could be viewed in real time. Selected key personnel in the research support and management team were given access to this password protected platform to view interview data, which were all de-identified, on the research console. This facilitated real time monitoring of data collection progress and data quality. Limited secured editing rights were given to the data manager and technician with recorded trail of data corrections. The technician was employed full time at an approximate cost of 20,000 USD per annum. The Mobenzi systems permitted an export system of all interview data to excel, which was imported to a statistical package, STATA for data analysis.

### Paper-based data collection

Paper surveys, that included written instructions below each question on limits, skips and compulsory questions, were used as a default option if mobile phones were faulty and needed to be replaced or if the software on the phone had a technical glitch. Paper based surveys would be captured onto the mobile phone sometimes on the same day or at a later stage (depending on the reason for non-direct mobile capture). If it was still not possible for the data collector to capture the surveys, the data collector handed these paper based surveys to the supervisor at the monthly face to face meetings. The supervisor couriered these paper based surveys to the office-based data assistant who captured the interview data.

## Results

### Cross-sectional surveys

In the 2010, 2011–12 and 2012–2013 surveys respectively, data from 10,554, 10,071, and 15,496 interviews, and from approximately 186 variables per survey were successfully uploaded to 151 mobile phones, across 580 health facilities in 51 districts and nine provinces onto a secure web-based research management console. In the 2012–2013 224 (2%) paper-based surveys were administered. Fifty-four phones were replaced due to broken devices/faulty phone keypads/ battery problems, there were 170 software application errors including 53 partial downloads, 26 software reloading and 91 missing visit time point folders.

### Follow up component

Follow-up data included 10,536, 6-week interviews and 15,496 post-natal interviews. In addition, 1974 missed visit forms, 9287 participants contact information, 1002 suspensions, 224 software/technical and device support queries and 1172 data queries were also in the follow-up research console. Regular communication between the technician, field based staff and the software manager /developer with the service provider resolved duplicate entries, incorrect entries, typographical errors and participant withdrawals from the study.

### System related challenges

During the course of these surveys, seven main technical challenges were documented including: 1) data loss, 2) freezing of mobile devices, 3) missing folders at visit timepoints/or missing PIDs 4) partial download of surveys, 5) changes in the formatting of dates that seemed to occur when data were merged. 6) user-related errors (Table 2). The technical problems either arose because the software application had a technical problem (unknown to the data collector), or a faulty cell phone. These errors required the attention of a full time mobile data system manager, to respond to field-based queries in real time. Additionally, a strong data manager, and co-operation from field-based staff was needed to resolve field-based queries such as infant weight, date of birth or date of death.

**Table 2:** System-related challenges experienced during data collection with mobile phones

Challenge	Solution
System-related	
(1) Data loss - loss of data may be attributed to users exiting the application before completing the interview. These interviews were not located	Re-training of data collectors so the application is not exited before interview completion. Re-interview was conducted if the mother/ caregiver agreed
(2) Freezing of phones either due to a) device problem and/or b) user related	Device had to be rebooted and/or reset
(3) Partial downloads – user exits or interrupts while the software is being updated or user not checking for updates timeously. This affects the retrieving of a full questionnaire on display.	Bulk SMS reminders were sent to users on a weekly basis to check for updates on the Mobenzi application. Mobile phone sent to service provider to check if there was a problem with mobile phone. If mobile phone related, it was replaced. If not, software was reloaded.
(4) Missing folders or missing PIDs at scheduled visit timepoints Unable to proceed further while conducting interview.	Data collector had check for updates on mobile phones daily. Missing folders was when the data collector did not check for updates. The data collectors who defaulted where sent SMS reminders individually to check for updates. Missing PIDs-were reported immediately to the data assistant who investigates and re-assign PIDs to data collectors’ phone. If still unresolved, mobile device software was reloaded.
(5) Changes in the formatting of dates that seemed to occur when data were merged.	Web developer need to be consistent when setting up date formatting across all platforms e.g. dd/mm/yyyy or yyyy/mm/dd. Different formatting created a problem when merges and data import into statistical packages occurs eg. 12/03/2015 is read as 03/12/2015
User related	
The handset used for data collection was a basic Nokia 2330 which was a compact phone with a small display screen and keyboard. The users were retired nurses who had difficulty in viewing information as the screen print was light in colour and small keyboard operation was difficult for them.	Re-training of data collectors and correcting data errors immediately

### Comparison of mobile data collection system with paper-based data collection

Within the Mobenzi platform there was a system set up to measure duration of interviews denoted by “start time” and “end and time” which is linked to the device, the user, the date of interview, province, district and facility. (Table 3).

**Table 3:** Screenshot of system set up denoting time

Fieldworker	Date of Visit	Time Start	Time End	Province	District	Facility
xxxxxxxx	29–10-12	9:00:08	10:08:46	LP	lp Vhembe District	lp Matsa Clinic
xxxxxxxx	29–10-12	9:46:54	10:34:32	WC	wc City of Cape Town Metropolitan	wc Westlake Clinic
xxxxxxxx	29–10-12	9:55:59	12:15:40	EC	ec Chris Hani District	ec Ntsimba Clinic
xxxxxxxx	29–10-12	10:04:06	12:30:54	NW	nw Bophirima District	Dryharts Clinic
xxxxxxxx	29–10-12	9:44:39	11:21:21	EC	ec Oliver Tambo District	ec Mthatha Gateway Clinic
xxxxxxxx	29–10-12	12:32:12	1:24:20	WC	wc City of Cape Town Metropolitan	wc Guguletu Clinic
xxxxxxxx	29–10-12	12:40:28	1:57:50	GP	gp Ekurhuleni Metropolitan	gp Boksburg North Clinic
xxxxxxxx	29–10-12	9:45:24	11:06:33	LP	lp Capricorn District	lp Dikgale Clinic

^xxxxxx^Data collector’s names blocked out to maintain confidentiality

At the early stages of implementation (within the first 2 weeks), the average time spent on capturing surveys was 53.03 mins (SD: 2.77, range 47–60). Once a data collector became comfortable with the device, the average time spent on capturing surveys was 37.56 mins (SD: 3.47, range 30–45).

Administering a paper-based questionnaire was between 60 and 70 mins with an additional 45 min for electronic data capture.

In 2012–13, 1172 data errors were identified via the web-based console. The time taken to correct errors on the web was approximately 3 days. Depending on the nature of the query, it sometimes took approximately 1 week to resolve and correct electronic data errors. This was due to the data collectors work schedule and their proximity to the clinic of origin of the data error.

Paper surveys couriered to the office for capturing, were prioritised and completed on the day of arrival. Data queries emanating from this batch of captured data took about 4 weeks to identify, resolve and correct (dependant on the timing of their monthly face to face meeting). Table 4 summarises the comparison.

**Table 4:** Mobile data collection system vs paper-based data collection

	Administering of Questionnaires	Corrections of Errors
Direct mobile capture (facility level)	30 to 45 mins	3 to 7 days
Same day paper-based data capture and mobile data capture	60 to 70 mins + 45 min	
Central office paper-based data capture	1 to 4 weeks	1 to 4 weeks

## Discussion

While the mobile data collection system described above may seem to be complicated to set up i.e. software development, technical protocol and implementation, careful planning during set-up tailored the system to the project, ensuring that the system design facilitated data collection and aided the monitoring and supervision of data collectors. The increased sophistication of the system was the result of lessons learnt during previous smaller scale studies on infant feeding and PMTCT conducted by members of this research team, where we identified gaps in monitoring, supervision and data quality. The use of technology in any research setting does have its challenges. The major challenge identified in studies that used mobile phones for data collection was the lack of mobile network coverage [10, 13]. This was not a problem in this national South African study as mobile network coverage is high in urban and rural South African settings. Concerns have previously been raised about data security when using mobile data collection systems [13, 14]. In this study there was no evidence of a breach in the security and confidentiality of data. Mobenzi developed a multi-layered security protocol that ensured a high degree of participant confidentiality throughout the study. Also, each study personnel signed a confidentiality agreement and there were no breaches. A major challenge highlighted in literature [11] is the size of phone. In this study older aged users had difficulty in viewing the small screen and operating a small keyboard resulting in data errors. Other challenges cited in low-middle income settings include theft or loss of research mobile phones, resulting in replacement costs escalating [15]. This was not experienced during these studies, which we hypothesise may have been due to the choice to use cheaper phones which were less attractive for theft.

Short message service (SMS) was a particularly useful application that was used to collect or share information and to enhance communication between health personnel and patients in a low-cost manner [16]. Use of SMS as a data collection tool was reported as feasible for delivery of information in real time, to improve information quality, reduce data losses and reporting errors and reduce data uploading difficulties [2]. In this study the SMS’s sent to participants were reminders that were effective and appeared to increase participant adherence to study visits.

Focused and accurate data collection increases study efficiency [6]. When properly designed, MDC solutions can offer a convenient cost effective approach for data entry, data management and reporting [6]. A well-designed electronic data collection system that is introduced with care amidst work processes that are adjusted to meet the study needs, will lead to time efficiency, more accuracy and cost-efficiency than the standard paper based data collection method [6]. However, there are weaknesses and challenges that must be considered and addressed for an MDC system to fulfil its potential.

## Conclusion

Our experiences demonstrate the feasibility of using mHealth during large-scale national surveys, in the presence of a supportive data management team. mHealth systems reduced data collection time by almost 1.5 times, and thus reduced data collector costs and time needed for data management. Although system set-up, design, testing and implementation required time and financial resources, including a fulltime technician to trouble shoot queries from the field, it reduced the time spent on data entry and cleaning and the time to dissemination of results to key stakeholders, thus the benefits outweighed any disadvantages.
